# Crystal Structure of Spy0129, a *Streptococcus pyogenes* Class B Sortase Involved in Pilus Assembly

**DOI:** 10.1371/journal.pone.0015969

**Published:** 2011-01-11

**Authors:** Hae Joo Kang, Fasséli Coulibaly, Thomas Proft, Edward N. Baker

**Affiliations:** 1 Maurice Wilkins Centre for Molecular Biodiscovery, University of Auckland, Auckland, New Zealand; 2 School of Biological Sciences, University of Auckland, Auckland, New Zealand; 3 School of Medical Sciences, University of Auckland, Auckland, New Zealand; Griffith University, Australia

## Abstract

Sortase enzymes are cysteine transpeptidases that mediate the covalent attachment of substrate proteins to the cell walls of Gram-positive bacteria, and thereby play a crucial role in virulence, infection and colonisation by pathogens. Many cell-surface proteins are anchored by the housekeeping sortase SrtA but other more specialised sortases exist that attach sub-sets of proteins or function in pilus assembly. The sortase Spy0129, or SrtC1, from the M1 SF370 strain of *Streptococcus pyogenes* is responsible for generating the covalent linkages between the pilin subunits in the pili of this organism. The crystal structure of Spy0129 has been determined at 2.3 Å resolution (*R* = 20.4%, *R*free  = 26.0%). The structure shows that Spy0129 is a class B sortase, in contrast to other characterised pilin polymerases, which belong to class C. Spy0129 lacks a flap believed to function in substrate recognition in class C enzymes and instead has an elaborated β6/β7 loop. The two independent Spy0129 molecules in the crystal show differences in the positions and orientations of the catalytic Cys and His residues, Cys221 and His126, correlated with movements of the β7/β8 and β4/β5 loops that respectively follow these residues. Bound zinc ions stabilise these alternative conformations in the crystal. This conformational variability is likely to be important for function although there is no evidence that zinc is involved *in vivo*.

## Introduction

Sortases are membrane-associated cysteine transpeptidase enzymes, best known for their role in the covalent anchoring of surface proteins to the cell walls of Gram-positive bacteria [1], [2]. This facilitates the proper display of such proteins, many of which play important roles in bacterial infection and pathogenesis. Mutant strains lacking sortase genes are attenuated in virulence in animal models of several bacterial infections [3], [4], [5], leading to the recognition of sortase enzymes as promising new therapeutic targets [6], [7].

Substrate proteins destined for sortase-mediated surface display possess common features at their C-termini; a sortase-recognition sequence motif LPXTG, or variations, followed by a stretch of hydrophobic residues and a short tail of positively charged amino acids. These features are collectively referred to as the cell wall sorting signal (CWSS) [2]. The hydrophobic region and charged residues impede translocation and secretion of the protein across the cytoplasmic membrane, allowing the membrane-associated sortase to recognise the sortase-recognition motif [8], [9]. The active site cysteine then cleaves the substrate protein between the Thr and Gly residues of the sortase-recognition motif, and an amide bond is formed joining the newly-formed C-terminal Thr carboxylate to an amino group from the cross-bridge of the cell wall peptidoglycan precursor. The protein, covalently linked to the cell wall, is subsequently displayed on the bacterial surface, facilitating functions in host-pathogen interaction [10], [11].

Sortases may be recognised by a characteristic sortase signature motif, TLXTC (X for any amino acid), in which the cysteine is the catalytic residue in the active site [12]. Searches of genome sequences point to a large and diverse family of putative sortases [13], which have been classified into four major groups, A, B, C and D [14]. It is increasingly clear that sortase function is not limited to cell wall anchoring. Whereas in many Gram-positive organisms a single “housekeeping” sortase anchors the majority of surface proteins to the cell wall, accessory sortases also exist that are dedicated to more specific functions by acting on small numbers of substrate proteins. The housekeeping sortases are generally class A sortases, whereas accessory sortases are spread among the remaining three classes [14]. Accessory sortases generally differ in specificity, recognising a different sortase recognition motif, and their genes tend to be co-located with their substrate proteins. For example, the class B sortase from *Staphylococcus aureus* recognises a motif NPQTN, and anchors the heme-binding IsdC protein to the cell wall; the *srtB* and *isdC* genes are located together in the *isd* iron-acquisition operon [15], [16].

In recent years, certain accessory sortases have been shown to be critically involved in the assembly of elongated surface appendages called pili for surface display on Gram-positive bacteria [17], [18], [19]. These pilus-specific sortases are encoded in the same gene cluster as the genes for their substrates, the pilus subunit proteins (pilins). The pilus backbone is formed from multiple copies of a single major pilin subunit, which are covalently linked like beads on a string by a pilus-specific sortase. Deletion of the gene for this sortase abolishes pilus polymerisation [17], [20], [21], [22], [23]. The mechanism of sortase-mediated pilus polymerisation is believed to be similar to that of cell wall anchoring, except that the C-terminal Thr carboxylate generated by sortase cleavage is then linked to the ε-amino group of a lysine residue from the next pilin subunit [17]. Finally, a housekeeping sortase transfers the pilus polymer to the cell wall precursor, in a manner analogous to the sortase-mediated cell wall anchoring of other surface proteins [24].

Structural analyses of representative sortases from classes A, B and C show that they share a common catalytic domain, based on a conserved β-barrel core [29], [30], [31], [32], [34], [35]. Sequence alignments, structural data, mutagenesis and activity assays point to conserved Cys, His and Arg residues with key roles in catalysis [25], [26], [27], [28], [29], [30]. Current mechanisms [25], [31], [32] involve nucleophilic attack by the thiolate sulfur of the essential Cys on the carbonyl carbon of the Thr residue of the LPXTG sorting signal, followed by bond cleavage and formation of a stable thioacyl intermediate. This intermediate is then resolved by nucleophilic attack on the thioacyl carbon by an unprotonated amino group from a second substrate. This may be either an amino group from the peptidoglycan of the cell wall, for cell surface anchoring, or a lysine ε-amino group from another pilin subunit, for pilus assembly. The His residue is believed to have a dual acid/base role, donating a proton to the leaving amide nitrogen during the cleavage reaction and accepting a proton from the amino group of the second substrate to allow nucleophilic attack by the unprotonated amine. The Arg side chain is implicated in substrate binding and possibly in stabilisation of a presumed oxyanion intermediate [25].

Important questions concerning specificity remain, however. Sortases recognise not only a particular C-terminal sequence motif on the substrate protein but also a specific amino group on the acceptor to which transfer occurs. The loops and helices that decorate the core β-barrel vary considerably among the different sub-families and play an important part, as is shown by protein engineering studies on *S. aureus* SrtA. Substitution of the β6/β7 loop region of SrtA by the equivalent region of SrtB changes the preferred substrate sequence motif from LPXTG, characteristic of SrtA, to NPQTN, characteristic of SrtB [33]. Interestingly, however, the engineered SrtA cannot efficiently complete the reaction by transfer to the preferred SrtB acceptor, implying that there are further recognition elements.

Thus far, the only pilus-specific sortases to be characterised structurally are the class C sortases SrtC1, SrtC2 and SrtC3 from *Streptococcus pneumoniae*, which all appear to be involved in the pilus assembly. These three sortases show very similar structural features, notably in the presence of a “lid” that covers the active site and is proposed to play an important role in the substrate specificity; differences are seen between the lids of SrtC1 and SrtC2, which contain a patch of positively charged residues, and that of SrtC3, which contains exclusively hydrophobic or negatively charged residues [34], [35].

Here, we focus on Spy0129, an accessory sortase responsible for assembly of the covalent polymeric shaft of the pili expressed by *Streptococcus pyogenes* (group A streptococcus; GAS) serotype M1. GAS pili mediate adherence to human tonsil epithelia, skin keratinocytes and pharyngeal cells during GAS infection [20], [36], and contribute to GAS virulence [20], [37]. Spy0129 is encoded in the same operon as the genes for three pilins: the major pilin Spy0128, the adhesin Cpa and the basal pilin Spy0130. Also encoded in this operon are a putative signal peptidase-like protein SipA, and a class C sortase of unknown function, Spy0135 [23], [38]. Spy0129 is the only pilus-specific sortase of GAS M1, having been shown by gene knockout to be essential for the polymerization of Spy0128 [20], [23]. In this process, Spy0129 cleaves between Thr and Gly of the sortase-recognition motif EVPTG of Spy0128, and links the Thr carboxylate *via* an isopeptide bond to the ε-amino group of Lys161 of the next major pilin subunit [39].

We have determined the crystal structure of Spy0129 at 2.3 Å resolution and show that it belongs to the class B sortase family, in contrast to the other characterized pilus-specific sortases that belong to class C. Accordingly, Spy0129 has structural features that differentiate it from other sortases, and in particular from the SrtC pilin polymerases from *S. pneumoniae* [34], [35]. This is consistent with the ability of Spy0129 to recognize a distinct sequence motif EVPTG and to transfer the substrate protein to a lysine acceptor that is displayed in a different structural setting, when compared with the lysine acceptors of the other major pilins.

## Results

### Structure determination

The Spy0129 structure solved in this study comprises the complete 237-residue polypeptide, with the exception of the first 35 residues, which constitute the transmembrane anchor and were omitted from the construct (Spy0129_36–237_) to permit soluble expression. Spy0129 was crystallized in the space group *I*4_1_22, with two molecules in the asymmetric unit. Crystals could only be obtained in the presence of 0.2 M Zn^2+^ and the asymmetric unit was found to contain 19 bound Zn^2+^ ions, which were used to derive initial phases by single wavelength anomalous diffraction (SAD) methods. The structure was subsequently refined at 2.3 Å resolution (R = 20.4%, R_free_ = 26.0%; see Table 1 for full details). For each of the two independent Spy0129 molecules, 16 residues at the N-terminus (residues 36–51) were not visible in the crystal structure and are assumed to be disordered, together with four additional N-terminal residues, GPGS, that resulted from the cloning. The two monomers are almost identical, except for some variations in the active site regions (see below). When the two molecules are superimposed, the root-mean-square (rms) difference in Cα atom positions is 0.95 Å over 169 aligned residues.

**Table 1:** Data collection, structure determination and refinement statistics.

Space group	*I*4_1_22
Unit cell dimensions (Å)	a = b = 94.51, c = 253.52
**Data collection statistics**	
Resolution range (Å)	50–2.32 (2.40–2.32)
Wavelength (Å)	0.97977
Total reflections	252425
No. unique reflections	25357 (2472)
Redundancy	10.0 (10.2)
Completeness (%)	99.8 (100.0)
Mean I/σ	33.2 (4.0)
R_merge_ (%)	6.5 (67.4)
**Structure determination**	
No. of heavy atom sites	17
Cullis R	0.84
Phasing power (anomalous)	1.04
FOM (acentric/centric)	0.36/0.16
**Refinement statistics**	
Resolution range	30–2.32
No. of reflections	23960
R_work_/R_free_	20.4/26.0
rms deviations – bond lengths (Å)	0.015
rms deviation – bond angles (deg)	1.52
No. of protein atoms	3090
No. of water molecules	189
Other species	19 Zn^2+^, 4 ethylene glycol, 5 acetate, 2 Cl^−^
Ramachandran plot – most favoured (%)	89.3
Ramachandran plot – outliers	0

a Values in parentheses are for outermost shell.

### Spy0129 has the canonical sortase fold with variations

**Figure 1:**
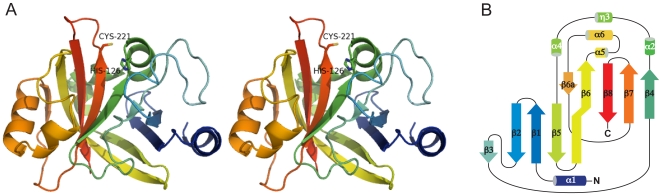
Structure of Spy0129. A) Stereo view of the monomer (molecule B), colour coded from N-terminus (blue) to C-terminus (red). The putative catalytic residues Cys221 and His126 are shown in stick representation. B) Topology diagram for Spy0129, coloured as in A) and labelled from α1 to α6 for α-helices and β1 to β8 for β-strands.

The overall structure of Spy0129 conforms to the archetypal sortase fold, in which a highly-twisted, 8-stranded β-sheet folds over to generate a core β-barrel, whose surface is decorated with loops and helices (Figure 1). The β-strands are predominantly antiparallel, but β4 is parallel to β7 and β3 is parallel to β2. A striking feature of the β-barrel is the curved surface formed by β-strands belonging mostly to the C-terminal half of the molecule, in the order β4-β7-β8-β6. This presents a large, concave surface, which provides the active site (see below) and is partly covered by α-helices from the β6/β7 loop. The long strand β6 contains a kink that enables it to hydrogen bond to β5 to form a second half of the β-barrel comprising β6-β5-β1-β2, and the short β3 strand hydrogen bonds to both β2 and β4 to complete the barrel (Figure 1).

Whereas the central β-barrel is well conserved in different sortases, the connecting loops and helices vary widely. In Spy0129, a 3-turn N-terminal α-helix (α1) leads into the first two strands of the β-barrel, β1 and β2, and packs against them. The most distinctive features of the Spy0129 structure, however, are the three long connecting loops β2/β3 (residues 86–105), β4/β5 (residues 126–150) and β6/β7 (residues 173–214), which between them shape the presumed substrate-binding region. The first part of the β2/β3 loop covers strand β3 and part of β4, and is adjacent to the putative catalytic histidine, His126. The β4/β5 loop begins with His126 and continues with three short helices α2, η3 (a 3_10_-helix) and α4 that close off one end of the barrel. This loop evidently has some flexibility, since the residues corresponding to helix α2 in monomer A form a loop instead in monomer B, changing the position of His126.

The β6/β7 loop provides the most notable difference between Spy0129 and other sortases. This long loop (42 residues) contains two further α-helices, α5 and α6, that cover much of the large concave face of the C-terminal half-barrel (Figure 1), shielding the mainly non-polar residues from solvent. There are slight differences between the two monomers that imply an inherent flexibility; residues 185–189, which belong to helix α5 in molecule A could not be modelled in molecule B due to ambiguous electron density. The β6/β7 loop also has an additional β-strand (β6a), unique to Spy0129, which hydrogen bonds to β6 to extend the concave surface of the C-terminal half-barrel.

### Spy0129 is a family B sortase

**Figure 2:**
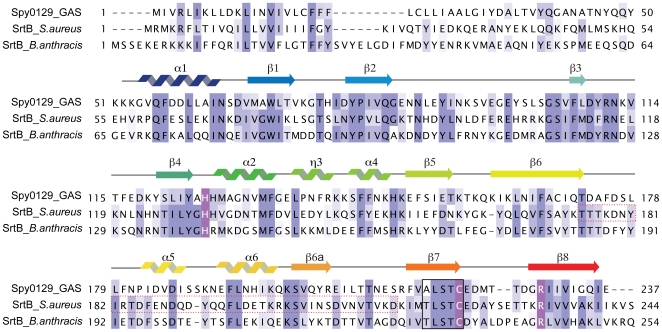
Sequence alignment of Spy0129 with other class B sortases. Invariant residues are highlighted in dark blue and conserved residues in lighter blue colours. Putative catalytic residues are coloured in purple. The secondary structure elements from Spy0129 are shown above the sequence. Residues corresponding to the sortase signature motif are boxed with a black outline. The β6/β7 loop region of *S. aureus* SrtB which was shown to confer substrate specificity is outlined with a dotted red box.

Sequence comparisons have previously identified Spy0129 as a class B sortase, on the basis of several sequence inserts that are specific to class B. Searches of the Protein Data Bank (PDB) with DALI [40] or SSM [41] confirm that the only structural homologues of Spy0129 are other sortases, and that its closest relatives are the SrtB enzymes from *Staphylococcus aureus* and *Bacillus anthracis* (SaSrtB and BaSrtB, PDB codes 1NG5 and 1RZ2)[30], [42]. Sequence identity with the two SrtBs is ∼32% (Figure 2), and their structures can be superimposed on to Spy0129 with a root-mean-square-difference (rmsd) in atomic positions of 1.76 Å for 163 equivalent Cα positions. In contrast, the SrtA enzymes from *S. aureus* and *S. pyogenes* (SaSrtA and SpySrtA, PDB codes 1T2P and 3FN5) [26], [32], match much less well, with sequence identity <20% and rmsds of 2.16 Å (108 Cα) and 2.03 Å (116 Cα) respectively. Similarly the three SrtC enzymes from *S. pneumoniae* [34], [35], two of which have pilin polymerase activity, all have sequence identities with Spy0129 of ∼15% and rmsds of 2.2–2.3 Å for 120–125 Cα atoms.

**Figure 3:**
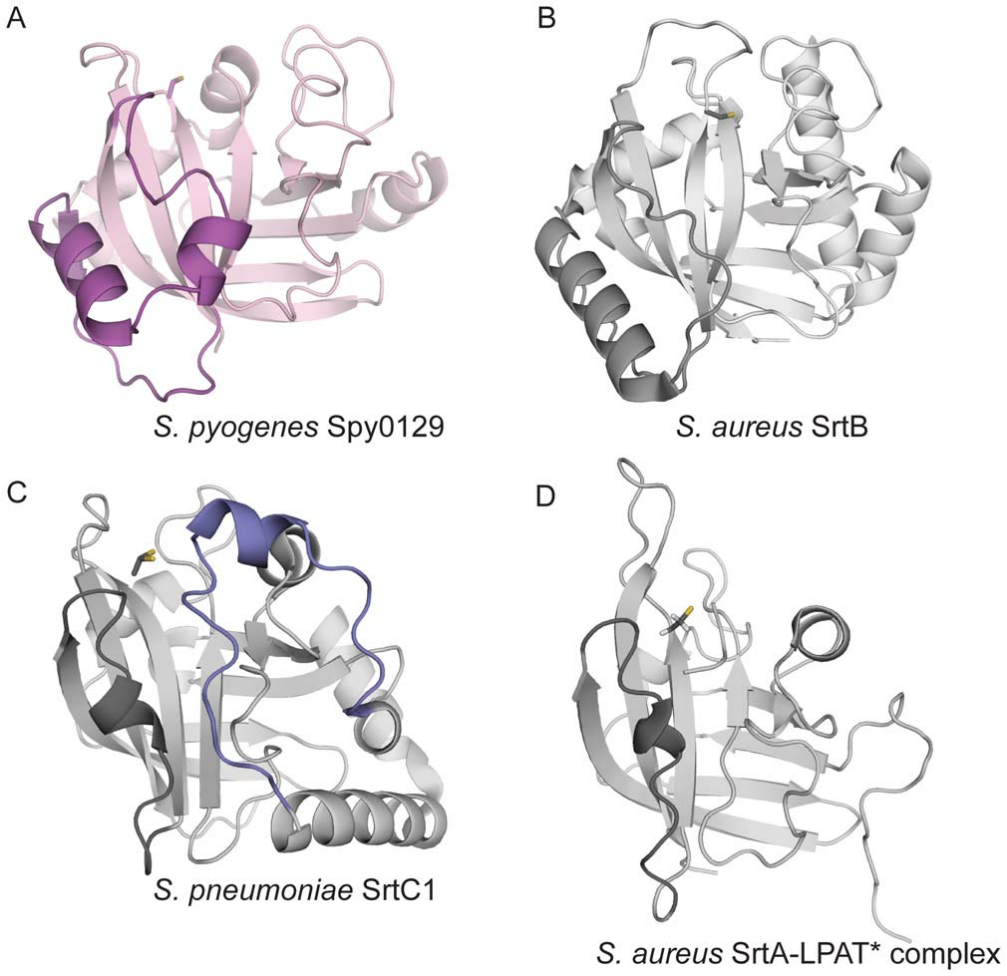
Comparison of different sortase structures. A) Class B: Spy0129 from *S. pyogenes* (this work); B) Class B: SrtB from *S. aureus* (PDB code 1T2P): C) Class C: SrtC1 from *S. pneumoniae* (PDB code 2W1J); and D) Class A: SrtA-LPAT* complex structure from *S.aureus* (PDB code 2KID). In each case, the β6/β7 region is highlighted with darker colour than the rest of the molecule. The lockable lid in the pneumococcal SrtC1 is highlighted in blue. All four structures are shown in equivalent orientations. The catalytic Cys residues are shown in stick mode.

All of the sortases share the same core β-barrel, with differences confined to the loops and helices that decorate it (Figure 3). Compared with the class A and C enzymes, Spy0129 and the two other class B enzymes have major insertions in the β2/β3 loop (11 residues), the β4/β5 loop (6 residues) and the β6/β7 loop (27 residues), together with differences at the N- and C-termini. Spy0129 differs from the other class B enzymes primarily at the N-terminus, where it lacks the first of the two helices found in BaSrtB and SaSrtB. In all sortases, conformational differences are common in the flexible parts of some surface loops, notably β4/β5, β6/β7 and β7/β8, where there are also deviations between the two independent Spy0129 molecules. These are discussed in the following sections and may relate to differences in substrate specificity.

### Structural flexibility in the catalytic site

**Figure 4:**
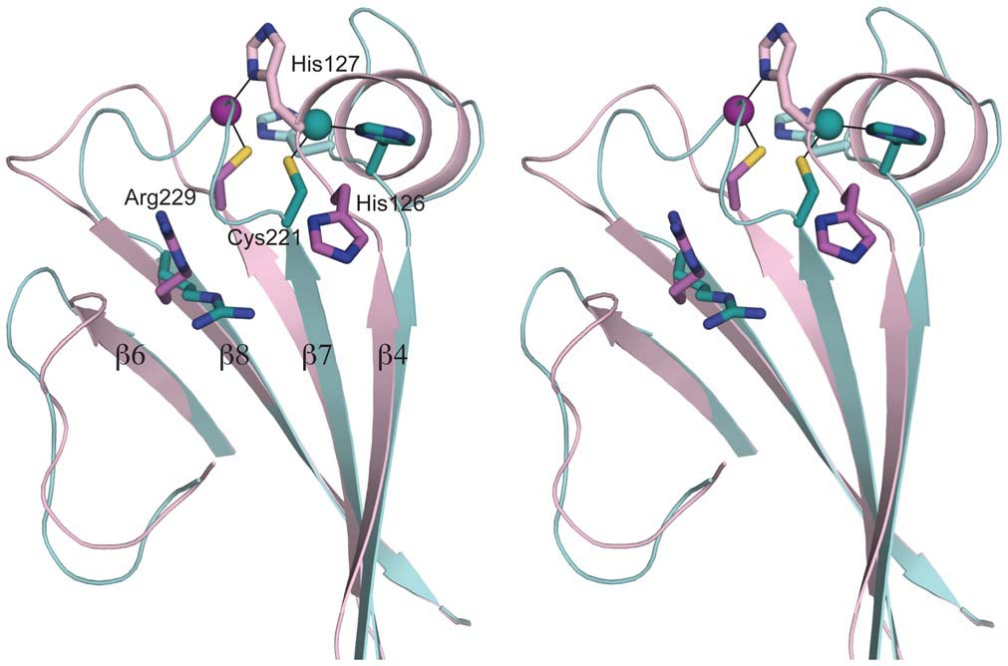
Active site of Spy0129. Stereo view of the active site region, with molecule A (magenta) superimposed on to molecule B (light teal) to show the conformational differences in the β4/β5 and β7/β8 loops. The residues Cys221, His126, His127 and Arg229 are shown in stick mode, coloured to correspond to the molecule to which they belong. In molecule A, Cys221 is linked to His127 through a bound zinc ion (magenta sphere) and His126 is oriented away from Cys221. In molecule B, Cys221 is linked to His126 through a zinc atom (blue sphere) and His127 is oriented away.

Previous studies on sortases indicate that a Cys-His dyad catalyses the transpeptidase reaction assisted by a conserved Arg residue that may be involved in orienting the substrate and/or stabilising the transient intermediate [6], [25], [27], [28], [32], [35]. These residues are strictly conserved in all sortases known to date and correspond with Cys221, His126 and Arg229 in Spy0129. As in the other sortases, these residues are located at the ends of three neighbouring β-strands from the C-terminal β-sheet of Spy0129; Cys221 is at the tip of strand β7, His126 at the tip of strand β4 and Arg229 at the beginning of the antiparallel strand β8 (Figure 4).

Distinct differences are seen in the active sites of the two independent Spy0129 molecules in the crystal. These differences, which involve the catalytic residues, are associated with conformational changes in the β4/β5 and β7/β8 loops that follow the His and Cys residues respectively (Figure 4). Alternative positions of these loops are stabilised by bound zinc ions that coordinate the active site residues in different ways in the two molecules, and by crystal packing. It is likely that the requirement for zinc for crystallization of Spy0129 results from its ability to rigidify these surface loops and thus permit stable crystal packing.

Comparisons with other sortase structures suggest that the structural variations seen in the active site do reflect real conformational variability that could be functionally important. The two conformations of the β7/β8 loop begin to diverge at Ala220, just prior to Cys221, and result in a deviation of ∼4.0 Å in the Cys221 Cα position (2.4 Å in Sγ). Superposition of Spy0129 on to other sortases shows that the molecule A conformation of the β7/β8 loop corresponds closely to that seen in class A and class C sortases, such as SpySrtA, SrtC1, SrtC2 and SrtC3, whereas that in molecule B more closely follows the course of the equivalent loop in the class B sortases. Flexibility is also seen in the β7/β8 loop of SaSrtA [29], [31], and we conclude that the variations in this loop and in the position of Cys221 reflect inherent conformational flexibility.

Identification of the catalytic histidine in Spy0129 is complicated by the fact that although sequence alignment with other sortases points to His126, a second histidine, His127 follows immediately after. Structural superpositions, however, show that in molecule B of Spy0129 the conformations of β4 and the β4/β5 loop match the equivalent structures of all other sortases, and that His126 superimposes on the catalytic histidine in each case. In this molecule, His126 is directed towards Cys221, with a bound zinc ion bridging between Cys221 Sγ and His126 Nδ1, whereas His127 projects into solution (Figure 4). We conclude that His126 is the catalytic histidine in Spy0129. Intriguingly, in molecule A, residues 126–133 form an α-helix, His126 is reoriented away from the active site and His127 instead is bridged to Cys221 Sγ by a bound zinc ion. Although this α-helical conformation at the start of the β4/β5 loop has not been seen in any other sortase to date, its B-factors are lower than for the molecule A conformation (40 Å^2^ compared with 49 Å^2^), suggesting that it represents a stable alternate structure. An NMR study of *B. anthracis* SrtA (BaSrtA) [43] also suggests flexibility in this loop, with resonances absent for Asn127, equivalent to His127 of Spy0129.

Although the zinc binding in the crystal may not be biologically relevant, the interaction between Cys221 and zinc in Spy0129 is consistent with the ability of this residue to form a thiolate nucleophile. The zinc bridging between Cys and His residues in the active site also maintains a common theme in sortase structure and function, in which these residues are not close enough to interact directly, being commonly ∼5 Å apart. Bridging by bound water is seen in the pneumococcal SrtC structures [35] and in SpySrtA, in which the active site Cys is only 2.16 Å away from a presumed water molecule that also bridges to the His residue [32]. Water bridging between the Cys and His residues has been suggested as a mechanism for transfer of the proton from Cys to His, allowing the latter to carry out its supposed role of protonating the substrate leaving group and facilitating intermediate formation [35].

### Substrate binding region

Data from a variety of sources identify a groove adjacent to the catalytic cysteine as the binding site for the LPXTG sortase recognition motif on substrate proteins. The most direct information comes from the NMR structure of a SaSrtA-substrate analog complex [31]. This is independently supported by spectroscopic and mutational studies on SaSrtA[29], [31], [44] and by modeling studies on SpySrtA[32]. The floor of the binding groove is formed by strands β4 and β7, with the walls contributed by a short 3_10_ helix from the β6/β7 loop on one side and parts of the β2/β3 and β3/β4 loops on the other. Another likely binding determinant, specific to the class C sortases, is a flexible lid that emanates from the N-terminal part of the SrtC enzymes, preceding strand β1, that folds over the active site [34], [35].

**Figure 5:**
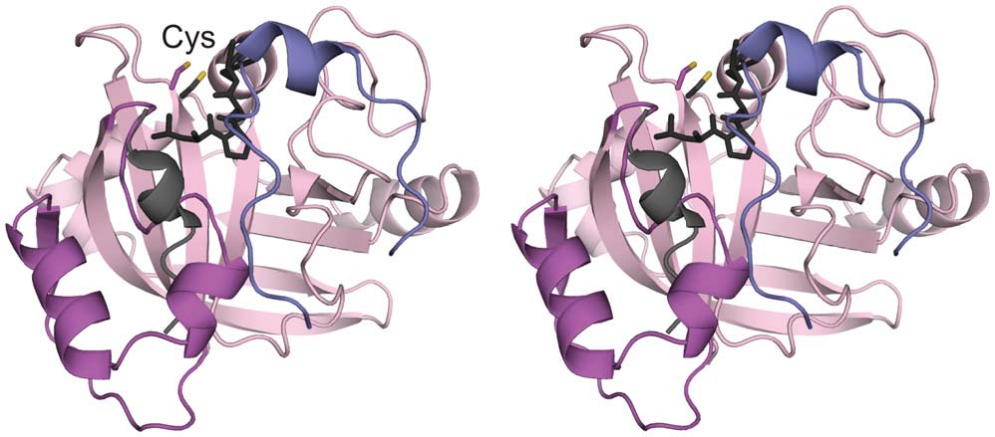
Surface structures involved in substrate binding. Stereo diagram of Spy0129 (magenta), with its β6/β7 region highlighted in darker magenta. Superimposed on to Spy0129 are the lockable lid present in the class C SrtC enzymes from *S. pneumoniae* (dark blue) and β6/β7 region from *S. aureus* SrtA (dark gray) including the 3_10_-helix that helps bind the LPAT* peptide analogue.

The surface loops in Spy0129, as in the other class B sortases, are very different from those in the class A and C enzymes (Fig. 3). Even though Spy0129 functions as a pilin polymerase, like the SrtC enzymes from *S. pneumoniae*, it has no such lid, indicating that this feature is not universal among the pilus-specific sortases. The portion of the active site floor that is covered by the lid in SrtC1, SrtC2 and SrtC3 is much more open and exposed to solvent in Spy0129 (Figures 3 and 5).

In contrast to the class A and C sortases, the most prominent surface feature that helps define the Spy0129 active site is the β6/β7 region, located on the opposite side of the binding groove from the SrtC lid (Figure 5). This substructure covers a large part of strands β6a, β6 and β7 and is a feature of all class B sortases, being the site of the major class B-specific sequence insertion that characterises this sub-family[14]. Although similar in length, the β6/β7 region in Spy0129 shows different organisation compared with its SrtB homologues. In Spy0129, it consists of a loop, two helices α5 and α6 followed by a short strand β6a, and another loop, whereas the β6/β7 regions of SaSrtB and BaSrtB comprise a loop, a long α-helix, and another loop. The strandβ6a in Spy0129 is a novel feature that effectively divides the loop region into two parts and could allow them to move independently when substrates bind, and in a different way from the *S. aureus* and *B. anthracis* SrtB enzymes.

Differences in the loops that form the walls of the substrate binding groove in Spy0129 make it difficult to predict its likely binding determinants from comparison with SrtA. Nevertheless, residues 174–182 from the β6/β7 loop in Spy0129 occupy the approximate position of the 3_10_-helix that contacts the conserved Leu-Pro residues of the LPXTG peptide in SrtA structures (Figure 5). Spy0129 has hydrophobic residues that are analogous to some of those that contact the LP motif in SaSrtA: Phe107, Leu122, Leu179 and Phe 180 in Spy0129 are the approximate spatial equivalents of Ala92, Ala104, Val168 and Leu169 in SaSrtA, although the structures that support them are different. Several polar residues are also present in the Spy0129 binding groove that could account for its altered specificity; Tyr124 and Ser219 in Spy0129 replace Ala118 and Ile182 in SaSrtA.

### Activity assays and binding studies

**Figure 6:**
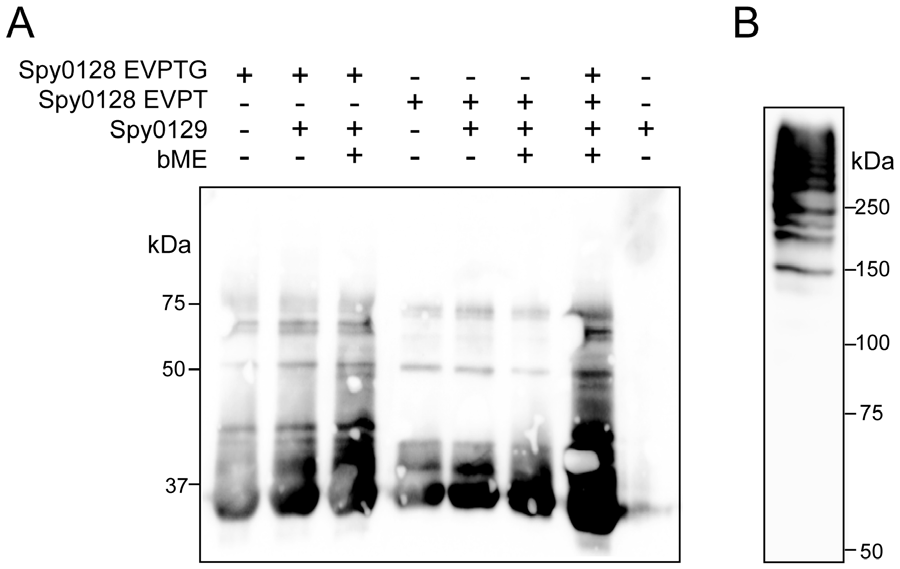
*In vitro* polymerisation assay. A) Immunoblot with polyclonal antibodies against Spy0128, after mixing Spy0128 and Spy0129 and incubating at 37°C for 20 h, showing the lack of a ladder of high molecular weight bands characteristic of polymer formation. B) Immunoblot of *S. pyogenes* M1 SF370 pili, composed mainly of polymerised Spy0128, probed with Spy0128 antibodies.

Several attempts were made to measure the catalytic activity. Firstly, we used a self-quenched fluorescent peptide 2-aminobenzoyl-KDFEVPTGVAM-diaminopropionic acid dinitrophenyl-NH_2_, mixed with various concentrations of Spy0129, to monitor substrate cleavage, following the methods used for detecting transpeptidase activity of cell-wall anchoring sortases [11], [45]. No cleavage could be demonstrated, however. Secondly, we used the same approach as that taken by Manzano *et al*. [34] for *in vitro* reconstitution of pneumococcal pili using SrtC1. The recombinant GAS major pilin Spy0128, containing the full sortase recognition motif, was mixed with Spy0129 at various ratios and incubated at 37°C for up to 20 h. Immunoblotting of these samples, however, showed no formation of high molecular weight bands that could represent pilus polymers (Figure 6). Attempts to co-crystallize peptides based on the sortase recognitioon motif, EVPTG, were also unsuccessful but are continuing.

### Bound zinc ions

Spy0129 could only be crystallized in the presence of 0.2–0.25 M Zn^2+^ ions. A total of 19 zinc sites were found to be associated with the two monomers of Spy0129 in the asymmetric unit, including seven intermolecular sites and one that is coordinated by water only. None of the Zn^2+^ ions occupy identical sites in the two molecules, and about half are coordinated by only one or no protein residues, an indication of weak binding. When the crystals were soaked in crystallization buffer free of Zn^2+^, 9 of the 19 Zn^2+^ ions were removed, including all those coordinated either by a single residue or by water only.

Analysis of purified recombinant Spy0129 by inductively coupled plasma-mass spectrometry (ICP-MS) (data not shown) showed negligible amounts of zinc, copper, calcium, iron and magnesium ions, and added metal ions did not activate the enzyme. We conclude that the bound Zn^2+^ ions in the crystal structure are probably not of functional importance, although they do play a role in stabilising alternate conformations of several loops and in stabilising the crystal packing. Interestingly, an active site mutant Spy0129 C221S was also produced, but could not be crystallized using ∼500 in-house crystallization conditions, including the same conditions as were used for the wild type. A possible reason is that the Zn^2+^ ions coordinated by Cys221 play an important role in crystallization of wild type Spy0129, by stabilising active site loops, and mutation of Cys221 to Ser would probably have eliminated this stabilisation by zinc.

## Discussion

Sortase enzymes are now understood to have critical roles in bacterial pathogenesis through their ability to covalently anchor substrate proteins to the bacterial cell wall, and to both assemble and anchor the pili expressed by many Gram-positive bacterial pathogens. Many of the proteins that are anchored are important virulence factors and/or are directly involved in colonisation and infection. This, together with the location of sortases on the bacterial cell surface, accounts for the strong interest in these enzymes for the development of new therapeutics.

The biochemistry of the sortase-catalysed transpeptidation reaction is becoming well understood, largely as a result of studies on the housekeeping class A sortase, SrtA [25], [31]. Although not all the details are worked out, key roles are played by the active site Cys residue (Cys184 in SaSrtA, Cys221 in Spy0129) which cleaves the Thr-Gly bond, with formation of a stable thioacyl intermediate, and the His residue (His120 in SaSrtA, His126 in Spy0129) which has an acid/base function, being able to donate and accept protons at different stages of the reaction [25], [31], [32]. In contrast, the structural basis by which sortases recognise their substrates is poorly understood, largely because of the difficulty of obtaining stable sortase-substrate complexes for structural analysis. The only direct information so far has come from the NMR structure of a complex between SaSrtA and a covalently-linked peptide substrate analog LPAT* that is attached to the catalytic Cys by a disulfide bond [31]. A SrtA-peptide complex crystal structure is available [29], but the peptide appears to be non-specifically bound [31].

Two recognition events are involved in sortase action. Firstly, the sortase recognition motif (LPXTG or a variant) of the substrate protein must be recognised and bound. Secondly, the acceptor substrate, to which the substrate protein will be transferred, must be recognised and bound, and a specific amino group brought into position to attack the thioacyl intermediate. Sortase-mediated pilus assembly provides an attractive system through which to address these issues since crystal structures of pilins can reveal the structural contexts of both the sortase recognition motif (the first substrate) and the resolving lysine residue (the second substrate).

Spy0129 has been identified as the pilus-specific sortase from the M1 SF370 strain of *S. pyogenes* [20], [23]. It assembles the polymeric pilus backbone, joining the Thr residue of the EVPTG sortase recognition motif on each Spy0128 major pilin subunit to the ε-amino group of Lys161 on the next Spy0128 subunit [39]. It also mediates attachment of the adhesin Cpa at the tip of the pilus [46] and attachment of the pilus shaft to the basal pilin Spy0130 [47], in each case through amide bond formation. The structure of Spy0129, presented here, confirms it as a class B sortase, closely related to the class B sortases from *S. aureus* and *B. anthracis* [30], [42], and sharing essentially the same surface loops and helices. It is, however, distinct functionally from the other class B enzymes, which anchor specific surface proteins containing an NPQTN motif to the cell wall [15], [16]. Spy0129 is also markedly different from the other known pilus-specific sortases, all of which appear to belong to class C. Members of this class have distinct and different surface structures, as seen in the *S. pneumoniae* SrtC enzymes [34], [35]. The class C enzymes also have C-terminal hydrophobic regions that are putative transmembrane anchors, whereas Spy0129 has only an N-terminal hydrophobic anchor. Any possible functional significance of this difference is unknown, however.

By analogy with SrtA, the β6/β7 loop region in Spy0129 seems likely to play a significant role in binding the sortase recognition motif of the first substrate. Substitution of this region in SaSrtA by the equivalent SrtB β6/β7 region is sufficient to confer a preference for the SrtB sorting signal [33]. In SaSrtA the backbone dynamics of this region change upon binding to Ca^2+^, which activates catalysis [48], and it has been shown to rearrange in the presence of the LPXTG substrate, forming a 3_10_-helix that helps generate a binding pocket with the underlying β-sheet [31]. Spy0129 has no equivalent 3_10_-helix, although it does have a spatially-equivalent portion of the β6/β7 loop, residues 174–182. On the other hand, despite its role in pilus polymerisation Spy0129 lacks any equivalent to the flexible lid of the class C enzymes. In its specificity, Spy0129 accepts either EVPTG from Spy0128 or VPPTG from Cpa as a sortase recognition motif. These sequences can also be QVPTG and VVPTG respectively in other *S. pyogenes* strains, implying a consensus (E/Q/V)(V/P)PTG sequence. Apart from the first position, the key residues are hydrophobic, suggesting that the hydrophobic residues on the β6/β7 loop, Leu179 and Phe180, plus Phe107 (β3) and Leu122 (β4) may be involved; there are also several nearby polar residues that could interact with the E/Q in position 1.

Crystal structures of the substrate proteins Spy0128 and Cpa show that the sortase recognition motif is in each case located on a peptide that extends only a short distance from the main body of their immunoglobulin-like (Ig-like) C-terminal domains [39], [49]. For both Spy0128 and Cpa, this Ig-like domain is stabilised by a covalent isopeptide cross-link just prior to the sortase recognition peptide. The substrate protein is thus highly unlikely to unfold when this peptide binds to Spy0129, implying that there is likely to be extensive contact between substrate protein and sortase in addition to the binding of the sortase recognition peptide. This contrasts markedly with emerging structural information on the basal pilin subunits for such pili, which are attached to the cell wall peptidoglycan by the housekeeping sortase SrtA. For these basal pilin subunits, the sortase recognition motif is typically located at the end of a long (35 Å), proline-rich, extended peptide that is well separated from the body of the protein [47].

These differences may explain the lack of *in vitro* activity for Spy0129. A short peptide carrying the sorting signal may be insufficient for productive binding and cleavage if additional interactions with the substrate protein are required. Importantly, gene knockout experiments have shown that, at least for the M3 strain of *S. pyogenes*, polymerisation of the pilus backbone does not occur in the absence of the signal peptidase-like protein SipA [50], implying that three proteins are required: major pilin, sortase and SipA. All three proteins are encoded together in the pilus operon, with SipA proposed to play a chaperone-like role in the pilus polymerisation [50]. The pilus operons of most other organisms do not include *sipA*-like genes, perhaps explaining why *in vitro* pilus assembly can be achieved for *S. pneumoniae* but not *S. pyogenes*. This phenomenon is not confined to Spy0129, however, since *B. anthracis* SrtA is also unable to carry out the transpeptidation reaction *in vitro* and may require an additional protein [43].

Why does *S. pyogenes* uses a class B sortase for pilus assembly, whereas all other Gram-positive organisms with similar pili, such as *S. pneumoniae*, *Streptococcus agalactiae* and *Corynebacterium diphtheriae* appear to use class C sortases? Structural data on the major pilins from *S. pyogenes* [39] and *C. diphtheriae* [51] suggest that it is the structural context of the acceptor lysine that is the key factor. All major pilins characterised to date share a common modular architecture comprising repeated immunoglobulin-fold (Ig-fold) domains. There are differences, however, in the numbers of domains and in the locations of the nucleophilic lysine residues. In the *C. diphtheriae* major pilin SpaA, the essential lysine, Lys190, is part of a YPKN pilin motif that is conserved in many other species [17] and is located on the last β-strand of the N-terminal domain of SpaA, close to the junction with the second domain [51]. For SpaA, as for the major pilins of *S. pneumoniae*, *S. agalactiae* and most other characterised species, a class C sortase mediates polymerisation. In contrast, the *S. pyogenes* major pilin Spy0128 is smaller than SpaA (two domains, compared with three), and the essential lysine, Lys161, is located in a different overall structural context, on a loop near the top of the N-terminal domain. The key lysine of the *S. pyogenes* basal pilin Spy0130 is similarly positioned [47], and neither of these proteins has the YPKN pilin motif.

We conclude that it is these structural differences in the *S. pyogenes* pilins that have resulted in the unique recruitment of a class B sortase for pilus assembly, compared with the class C sortase-catalysed polymerisation of the others. The co-location of pilin genes and their associated sortases is consistent with the ability to recruit varied sortase genes from the wider sortase family. These then become adapted to particular substrates, with the flexible lids in the SrtC enzymes [34], [35] simply providing an alternative structure for substrate selection compared with the extended β6/β7 regions used by class B sortases such as Spy0129.

Finally, the conformational differences in the β4/β5 and β7/β8 loops of the two independent Spy0129 molecules point to a flexibility that may be important for function. The active site histidine, His126 in Spy0129, is located at the start of the β4/β5 loop. This residue is believed to have a dual acid/base role, protonating the leaving group in the cleavage reaction and deprotonating the attacking amine in the transfer reaction [31]. The conformational change in Spy0129, from a loop in molecule A to a two-turn α-helix in molecule B, moves the His126 ∼5 Å and could enable it to carry out these two roles. The conformational change also brings the neighbouring His127 into the active site in molecule A, giving it a potential role in substrate binding or catalysis. Conformational flexibility is seen for this region in BaSrtA [43], and in both BaSrtA and SaSrtA the β7/β8 loop is also flexible and has been implicated as the likely binding site of the lipid II substrate [31],[43]. This loop is highly variable, in length and conformation, in different sortases, supporting its possible role in binding the second substrate.

### Conclusions

Spy0129 is critically involved in the pilus polymerisation in GAS serotype M1. The structure of Spy0129 presented here is the first for a class B sortase with a pilus-specific function. Although similar in functionality, Spy0129 does not share the unique features of the pneumococcal pilus-specific class C sortases, such as the lockable lids and the C-terminal transmembrane anchor. Instead, Spy0129 is more closely related to *S. aureus* and *B. anthracis* SrtBs, which are cell wall anchoring sortases. This demonstrates that pilus polymerising activity *per se* is not associated with unique structural features or a particular class of sortase, but is instead the result of the co-evolution of the pilin components with their cognate sortases to enable the appropriate substrate selection.

## Materials and Methods

### Protein production and purification

The *spy0129* gene was cloned using the conventional ligation method. DNA encoding amino acids 36–237 of Spy0129 (gi|13621429) was amplified from GAS serotype M1 genomic DNA using PCR with a forward primer 5′- GGATCCTTAACAGTTTATC AAGGAGC -3′ and reverse primer 5′- GAATTCTTATTCAATTTGTCCAATAAC -3′. The amplified product was cloned into the plasmid pET32-3C expression vector for His-tagged fusion protein expression [52]. The protein was overexpressed in *E. coli* BL21 (DE3) pRP cells in ZYP-5052 auto-induction medium [53]. Cells were grown at 37°C for 4 h followed by 20 h at 28°C before harvesting. Harvested cell pellets were resuspended in a lysis buffer containing 50 mM HEPES-NaOH pH 8.0, 200 mM NaCl and lysed using a cell disruptor (Constant Systems). The lysate was cleared by centrifugation for 30 minutes at 13,000 *g* prior to protein purification.

Lysate from a 500 ml culture was loaded on to a 5 ml HiTrap column (GE Healthcare) charged with Ni^2+^ and washed with 5 column volumes (CV) of lysis buffer followed by 5 CV of lysis buffer containing 50 mM imidazole. His-tagged Spy0129_36–237_ protein was eluted with 6 CV of 100 mM imidazole in lysis buffer. The His-tag was cleaved from the fusion protein by incubating the eluted protein with 400 µg of recombinant picornavirus 3C protease and 2 mM DTT overnight at 4°C. The DTT was then removed by dialysis and the protein solution was loaded on to a charged HiTrap column. The column was first washed with 2 CV of lysis buffer and then with 2 CV of 500 mM imidazole in lysis buffer. The flow-through and the first wash contained most of the untagged Spy0129_36–237_. The protein was further purified by size exclusion chromatography using a Superdex 200 HR10/30 gel filtration column (GE Healthcare) in buffer comprising 50 mM HEPES-NaOH pH 8.0 and 200 mM NaCl. Purified Spy0129_36–237_ contained 4 additional N-terminal residues that remained after the His-tag removal (Gly-Pro-Gly-Ser). An active site mutant containing Ser instead of Cys221 (Spy0129_36–237_ C221S) was generated using the overlap extension PCR method [54] using primers 5′- caacctctgaggatatgacaacag -3′ and 5′- cctcagaggttgataaggcaac -3′. The mutant protein was expressed and purified in the same way as described above for the wild type. The circular dichroism (CD) spectra of the mutant and wild type proteins were almost identical, indicating that the single residue mutation does not have noticeable effect on the secondary structure of Spy0129.

### Protein crystallization

Initial crystallization screens were carried out at 18°C in sitting drops in 96-well IntelliPlates (Art Robbins Instruments) with protein concentrations ranging from 6 mg/ml to 23 mg/ml. A condition comprising 10% (*w/v*) polyethylene glycol (PEG) 3350, 0.2 M Zn(OAc)_2_, 0.1 M NaOAc pH 6.2 produced diffraction-quality crystals. Typically, heavy precipitation first appeared in the drops and bipyramidal crystals grew after 3–7 days. Larger crystals were obtained in 1–4 µl hanging drops, using the same crystallization conditions and were used for data collection. Heavy precipitates tightly encased the Spy0129 crystals, which were removed using acupuncture needles when harvested for data collection. Fine screening around the original condition was carried out to see if such a high concentration of Zn(OAc)_2_ was required for the crystallization. Zinc acetate was found to be essential, with 0.2 M being about the minimum concentration required for crystallization; replacing Zn(OAc)_2_ with similar molar concentrations of salts containing other divalent metal ions such as Ca^2+^ and Mg^2+^ did not produce crystals.

### Data collection and structure determination

Spy0129 crystals were prepared for X-ray diffraction by brief immersion in a series of mother liquor solutions containing increasing amounts of ethylene glycol (up to 20%) followed by flash-freezing in liquid nitrogen. A complete set of single wavelength anomalous diffraction (SAD) data (Table 1) was collected at the Stanford Synchrotron Radiation Laboratory (SSRL) beam line BL9-1. The data were collected at 0.97977 Å, a wavelength remote from the Zn absorption edge, and indexed and scaled using the HKL2000 package [55]. The crystal was found to be in space group *I*4_1_22 with unit cell dimensions of *a* = *b* = 94.51 Å, *c* = 253.52 Å, α = β = γ = 90°, and contained two Spy0129 molecules in the asymmetric unit. The data were truncated to 2.32 Å due to the high *R*_sym_ in high resolution shells. The structure of Spy0129 was determined from single wavelength anomalous diffraction (SAD) data, using the program autoSHARP [56] to find heavy atom sites and carry out heavy atom refinement, density modification and solvent flattening. Seventeen Zn sites were located, giving initial phases with a figure of merit of 0.31/0.16 (acentric/centric). After density modification by SOLOMON [57], the figure of merit improved to 0.55/0.43 (acentric/centric). The phases generated from autoSHARP were submitted to ARP/wARP for autobuilding [58]. The resulting model covered 99% of total residues in the asymmetric unit with 341 out of 404 residues docked to the sequence. Further manual model building was carried out using COOT [59]. The model was refined using all data to 2.32 Å resolution using the program REFMAC [60]. Although the two molecules in the unit cell are related by 2-fold non-crystallographic symmetry (NCS), NCS restraints did not improve the refinement and hence were not used. In monomer B, three residues (V185, D186 and I187) were not modelled, as the electron density in this region was ambiguous.

An anomalous difference map was calculated by combining anomalous differences, (DANO, with standard deviation SIGDANO) with the final phases and FOM from autoSHARP to confirm the positions of the anomalous scatterers. This showed strong peaks for all the Zn sites found in autoSHARP, together with two additional Zn sites that were then added to the model. The *R* factor of the final model was 20.4% and *R* free 26.2%. About 90% of residues were in the most favoured regions of the Ramachandran plot with no outliers according to PROCHECK [61]. The atomic coordinates and experimental structural factors amplitudes for Spy0129 have been deposited with the Protein Data Bank, with accession code 3PSQ.

### *In vitro* polymerisation of Spy0128 by Spy0129

Spy0128_18–311_ (Spy0128 EVPT) and Spy0128_18–312_ (Spy0128 EVPTG) were cloned and purified as previously described [39]. Spy0128 (40 µg) and Spy0129 (80 µg) were mixed with or without 1% β-mercaptoethanol to a total volume of 50 µl and incubated for 20 hours at 37°C. The reactions were analysed on 12% SDS-PAGE gels followed by immunoblotting using rabbit polyclonal antibodies raised against Spy0128_18–308_. The blots were incubated with donkey anti-rabbit IgG conjugated to horseradish peroxidase (GE Healthcare) and immunoreactive proteins were detected by using chemiluminescent substrates (Perkin Elmer).

### Spy0129 transpeptidation assay

The transpeptidation assay using a fluorescence resonance energy transfer (FRET) substrate was designed based on the published protocols [11],[45]. In this assay, when a peptide flanked by a fluorophore and a quencher is cleaved by a sortase, the fluorophore is separated from the quencher and a concomitant increase in fluorescence is measured. The reactions were carried out in 200 ml in 96-well black-walled and clear-bottom plates at 37°C. Purified Spy0129 (542 µM) was mixed with a fluorescence substrate, 2-aminobenzoyl-KDFEVPTGVAM-diaminopropionic acid dinitrophenyl-NH_2_ (Abz-KDFEVPTGVAM-Dap(Dnp)-NH_2_), at concentrations ranging from 0 to 100 µM. The reaction was carried out in 50 mM Tris-HCl, pH 7.5, 150 mM NaCl and with and without 0.2 mM lysine. Fluorescence was measured using a SpectraMax microplate reader (Molecular Device) with excitation at 320 nm and emission at 410 nm. For endpoint measurements, the FRET substrate was mixed with 270 µM wild type or mutant Spy0129 and the fluorescence emission at 410 nm was measured before (T = 0) and after incubating the reaction for 3 hours (T = 3 hr) at 37°C.
